# Firefighters Are More Physically Active On-Duty Compared to Off-Duty

**DOI:** 10.3390/ijerph17249380

**Published:** 2020-12-15

**Authors:** Allison M. Barry, Katie L. Lyman, Nathan D. Dicks, Christi R. McGeorge, Michael J. Carper, Tanis J. Walch

**Affiliations:** 1 Department of Health, Human Performance, and Recreation, Pittsburg State University, Pittsburg, KS 66762, USA; mcarper@pittstate.edu; 2 Department of Health, Nutrition, and Exercise Sciences, North Dakota State University, Fargo, ND 58108, USA; katie.lyman@ndsu.edu; 3 Department of Nutrition, Dietetics, and Exercise Science, Concordia College, Moorhead, MN 56562, USA; ndicks@cord.edu; 4 Department of Human Development and Family Science, North Dakota State University, Fargo, ND 58108, USA; christine.mcgeorge@ndsu.edu; 5 Education, Health, and Behavior Studies, University of North Dakota, Grand Forks, ND 58202, USA; tanis.walch@und.edu

**Keywords:** firefighters, physical activity, BMI, obesity

## Abstract

Physical inactivity, coupled with increasing obesity levels, in firefighters plays a key role in aggregated cardiovascular events. The purpose of this study was to investigate device-measured physical activity (PA) for firefighters while on- and off-duty to have a clearer understanding of their overall PA level. Methods: Twenty-nine career firefighters participated in this non-experimental, within-subjects study by wearing an accelerometer to assess PA intensities and step-count. Obesity was classified using body mass index (BMI). Dependent *t*-tests were used to examine mean differences in PA intensities when on- and off-duty. Pearson product-moment correlations were used to assess the association between PA intensities when on and off-duty. Results: According to the World Health Organization BMI categorizations, 20 firefighters were overweight, 9 were obese, and, thus, none were normal weight. Only light PA (LPA) was statistically significant (*p* = 0.026) for on- and off-duty days with a small-to-medium effect size (*d* = 0.47), meaning that on average, firefighters performed more minutes of LPA when on-duty compared to off. There was a significant difference between on- (9060.2 ± 2636.4) and off-duty (7495.3 ± 2835.8) daily step counts (*p* = 0.011). Conclusion: As the results demonstrate, there is a dire need for increased PA levels in firefighters while on- and off-duty.

## 1. Introduction

Physical inactivity is currently the fourth leading cause of death worldwide, which classifies the condition as a global issue [1,2]. As the general public attempts to mitigate a rising obesity epidemic, firefighters are also exhibiting increased obesity prevalence within their ranks [3]. As is the case in the general public, excessive body weight in firefighters predisposes them to not only cardiovascular disease, but may lead to major cardiovascular events (e.g., myocardial infarction) while performing necessary job duties [3]. In the past 38 years, the number one contributor to on-duty firefighter fatalities was sudden cardiac death [4]. Although the overall number of on-duty deaths has declined, the risk of cardiovascular events remains relatively high.

The National Fire Protection Association (NFPA) developed codes 1582: Standard on Health-related Fitness Programs for Firefighters and 1583: Standard on Health-Related Fitness Programs for Fire Department Members to address the high rates of physical inactivity and obesity in firefighters across the United States (U.S.) [3,5,6,7,8,9]. Although the codes were implemented into the U.S. Fire Service, each departments’ adherence to these codes remain voluntary; therefore, it is the discretion of the administrative officials at individual departments as to whether or not they comply with the proposed standard. Currently, in the U.S., roughly one quarter of fire departments employ firefighters who engage in a basic fitness and health program. Unfortunately, this number has decreased 3% over the past eight years (NFPA Needs Assessment, 2015).

The development of health and wellness programs requires a considerable amount of time and financial resources. From the years 2011–2014, only 1% of all funding from the Federal Emergency Management Agency (FEMA) was allocated to improving health and wellness for firefighters in the U.S. (NFPA Needs Assessment, 2015). Therefore, firefighters may not be receiving external motivation from departmental administrators to maintain physical activity (PA) levels necessary to sustain the intense physiological demands experienced on the fire ground.

Few studies have investigated PA levels in firefighters to better understand how they can maintain cardiorespiratory fitness levels [6]. One study surveyed the frequency, intensity, and duration of PA of 527 career firefighters from the Midwest. Among those firefighters, roughly 25% subjectively reported they engaged in at least 150 min of PA per week [6], which meets the American College of Sports Medicine’s (ACSM) PA guidelines [10]. Thus, these researchers found that 75% of the firefighters in their study were not meeting the basic recommendations for PA, which raises concerns about these participants overall physical fitness, body mass index (BMI), and cardiovascular health. Surveys measuring PA are often influenced by social desirability bias in the form of over estimating the amount of PA individuals engage in [11]. To eliminate such potential biases, device-measured PA brings a level of objectivity to the data and the ability to precisely categorize PA intensity level.

The increased obesity levels, as well as an augmented risk of cardiovascular events while engaging in job-specific tasks, enhances the need for further investigation of PA in firefighters. Specifically, comparing PA levels while on- and off-duty will allow administrative personnel to focus their efforts on how best to encourage immediate lifestyle changes. Thus, it is important to investigate the PA intensities that firefighters engage in during their natural environment while on- and off-duty. The purpose of this study was to investigate device-measured PA for career firefighters in their natural environment, while on- and off-duty, to gain a clearer understanding of their overall PA levels.

## 2. Materials and Methods

Active, structural, career full-time firefighters (*n* = 30) were recruited from one U.S. Midwestern fire department. Participants were excluded from the study if they had musculoskeletal injuries precluding the firefighter from active, on-duty tasks. The affiliate university’s Institutional Review Board approved the study; written informed consent was obtained from each participant.

PA and anthropometric measures were assessed in a non-experimental, within-subjects design. Height was assessed using a portable stadiometer (Seco Corp., Model 213, Hamburg, Germany); weight was measured using a digital scale (Denver Instruments, Model DA 150, Bohemia, NY, USA). Body mass index (BMI) was defined as weight in kilograms divided by square of height in meters (kg∙m^−2^).

Firefighters wore an ActiGraph GT3X+ (ActiGraph Corp, Pensacola, FL, USA) accelerometer on their right hip via an elastic waist band for the duration of one tour, which consisted of: 24 h on-duty, 24 h off-duty, 24 h on-duty, 24 h off-duty, 24 h on-duty, and finally four days off-duty (9 days total). This tour format is specific to this department as tours vary from department to department in the U.S. The GT3X+ accelerometer uses microelectricomechanical accelerometers that use raw electrical signals and translates them, using proprietary algorithms, into values of movement. These data points are used to produce estimates of activity duration and intensity. Intensity of activity was classified as sedentary, light PA (LPA), moderate PA (MPA), moderate-to-vigorous PA (MVPA), and vigorous PA (VPA). The firefighters wore the accelerometer during waking hours except for water-based activities. Firefighters completed a sleep log detailing when they put on the accelerometer in the morning, and when they removed the accelerometer to go to bed with the intention that they would be asleep the whole night. Firefighters were required to wear the GT3X+ for at least four days, with a total of at least 10 h of wear time per day, to be included in the analysis [12,13]. The frequency of the GT3X+ was set at 30 Hz. The epoch duration was set at 60 s epochs [14]. In order to classify PA intensity levels, Freedson’s [14] cutpoints were used in conjunction with Choi’s non-wear time algorithm [15]. Mean daily accelerometer wear-time was also derived from the accelerometer data. To control for potential wear-time differences for on- and off-duty days, percentages were calculated to account for time spent in the different levels of PA intensities. ACSM’s PA guidelines were used to assess if firefighters met the minimum total amount of MVPA rather than using the 10 min bout increments while on- and off-duty. Tudor-Locke and Bassett (2004) step count indices were also used to categorically classify healthy adults into five categorical groups: (1) “sedentary behavior” is <5000 steps/day; (2) “low activity” without an exercise regimen is 5000–7499 steps/day; (3) “somewhat active”, with some intense activities including occupational demands, is 7500–9999 steps/day; (4) “active” is ≥10,000 steps/day; and (5) “highly active” is >12,500 steps/day [16].

Demographic characteristics were described using mean, standard deviation (±SD), and frequency. Dependent t-tests were employed to examine mean differences in PA intensities between on- and off-duty days within participants. Dependent t-tests were employed to examine mean differences in PA intensities as a percentage of wear-time. A dependent t-test was also employed to examine the mean differences in on- and off-duty step counts. Pearson product-moment correlations were employed to assess the association between PA intensities for both on- and off-duty days. In order to explore differences in PA intensities and step counts between firefighters who met and did not meet the ACSM guidelines, independent t-tests were calculated for both on and off duty time days. Analyses were performed in SPSS 24 (IMB Corp., Armonk, NY, USA). The level of significance was set a *p* < 0.05.

## 3. Results

A total of 29 out of 30 firefighters (age: 34.45 ± 7.15 year; BMI: 28.97 ± 2.52 kg∙m^−2^) completed all aspects of the study. One participant had to drop out of the study due to an unforeseen illness. According to the World Health Organization BMI categorizations, 20 firefighters were overweight, 9 were obese, and, thus, none were normal weight. Mean (±SD) accelerometer wear-time was 15.8 (1) h for on-duty days and 15.0 (1.3) h for off-duty days. There was a statistically significant difference for total wear time of the accelerometer between on- and off-duty days with a mean difference of 51 min (*p* < 0.05). After conducting a dependent *t*-test, only LPA was statistically significant (*p* = 0.026) for on- and off-duty days with a small-to-medium effect size (*d* = 0.47), meaning participants performed more minutes of LPA when on-duty (351.11 ± 59.90) compared to off (315.83 ± 86.90; see Table 1). The dependent *t*-test revealed that the difference in MVPA for on- and off-duty days that was trending towards significant, but was not statistically significant (*p* = 0.055). There was a small-to-moderate effect size (*d* = 0.40) as on average participants performed more minutes of MVPA while on duty. After the data was normalized controlling for total wear-time, there were no significant differences among PA intensity levels between on- and off-duty days (Table 1). Additionally, there was a significant difference between on- (9060.2 ± 2636.4) and off-duty (7495.3 ± 2835.8) daily step counts (*t* = 2.71, df = 28, *p* = 0.011). Specifically, 45% of participants met the standards for “active or highly active” for on-duty shifts based on the step count indices, compared to only 14% during off-duty shifts (Table 2).

Additionally, more firefighters met the ACSM PA guidelines during on-duty than off-duty (see Table 3 and Table 4). There was a significant difference between firefighters who met the ACSM PA guidelines compared to those who did not meet the ACSM PA guidelines for MPA, MVPA, and VPA during on-duty (Table 3) as well as off-duty days (Table 4). There was, however, a trend toward significance for LPA between those who met and did not meet the ACSM PA guidelines during on-duty shifts (*p* = 0.54, *d* = 0.77). After the data were normalized comparing for total wear-time, there was a significant difference between those who met and did not meet the ACSM PA guidelines for sedentary, MPA, MVPA, and VPA during on-duty days (Table 3). Comparatively, only MPA, MVPA, and VPA were significantly different between those who met and did not meet ACSM PA guidelines while off-duty (Table 4).

## 4. Discussion

The current investigation appears to demonstrate that firefighters were more physically active on-duty than off-duty (Table 1), with only a little over half of the firefighters meeting the ACSM PA guidelines while on-duty (Table 3). This investigation also demonstrates a significant difference in average MPA, MVPA, VPA, and step count between those firefighters who met and did not meet the ACSM PA guidelines for both on- and off-duty days (Table 2, Table 3 and Table 4). Additionally, 45% of firefighters in this study were classified as active or highly active according to step counts for on-duty compared to 14% when off-duty (Table 2).

Given that career, full-time firefighters, like most adults, spend the majority of their waking hours in an occupational environment, it is imperative to have a clearer understanding of occupational PA levels [17]. As a contrast with career firefighters, Thorp and colleagues (2012) explored PA in individuals with office jobs by providing an accelerometer to be worn during working and non-working days [18]. The results of the study demonstrated that individuals with office jobs engaged in sedentary activity for roughly 77% of their 15.3 h workdays compared to 63% during non-work days. Comparatively, the firefighters in the current study engaged in sedentary activity for roughly 60% of their on-duty shift and 61% of their off-duty day. Suggesting that firefighters engaged in less sedentary activities when on- and off-duty compared to office workers. However, when comparing the amount of time spent in MVPA, the difference between career firefighters and office workers basically disappears. In particular, the office workers in the Thorp et al. study spent 1.9% of their time in MVPA during the workday and 4.3% in MVPA on non-working days. Even though the firefighters participated in more MVPA during on-duty days (3.7%) than off-duty days (3.1%), the total amount of MVPA is essentially similar to that of office workers.

Although the comparison of firefighters to office workers may, at first, seem arbitrary, the results of the PA of firefighters is comparable to the subjects in the Thorpe study. Common logic would suggest office workers would have a high proportion of sedentary time. Although the current study demonstrates firefighters engage in a large amount of sedentary time, it should be highlighted that firefighters are expected to engage in physiologically demanding tasks with no advanced warning, which is very different from the average office worker. The sudden shift in physiologic responses (e.g., transitioning from a resting state to an active state), stimulates the sympathetic nervous system, resulting in a rapid increase in the firefighter’s heart rate and blood pressure [19,20]. The initial increase in heart rate remains constant throughout fire suppression tasks and may result in transient ST-segment changes, which are indicative of myocardial ischemia [19,21,22]. These physiological derangements can lead to potential myocardial infarctions resulting in sudden cardiac death [23]. In order to mitigate the chance of a cardiovascular event, more research is needed to study the effects of individualized wellness or fitness programs that prepare individuals for the sudden activation of the sympathetic nervous system.

High levels of sedentary activity could be an important cause for alarming rates of obesity in firefighters [3,6]. One study investigated the incidence of overweight and obesity in firefighters, compared to the general U.S. population, and demonstrated that roughly 76% of career firefighters are overweight or obese compared to 68% of the general U.S. population [3]. Additionally, a recent study demonstrated that 50% of firefighters had an increase in body weight gain (6.6 kg) and cardiovascular disease risk factors over a five-year period [24]. In the present study, no firefighters were considered normal weight based on BMI, which could pose a serious safety risk while both on- and off-duty. It has been demonstrated that augmented PA levels have been associated with decreased rates of obesity [25]. Therefore, the National Institutes of Occupational Safety and Health (NIOSH) Fire Fighter Fatality Investigation and Prevention Program has recommended the need to incorporate fitness and wellness programs to increase PA levels, which could potentially mitigate the obesity levels and adverse cardiovascular events in firefighters [26].

A strength of the current study was utilizing device-measured PA while firefighters were on- and off-duty. It is important to note that the PA measured while on-duty included all medical and emergency calls. Moreover, to the authors’ best knowledge, this is the first study to use device-measured PA with firefighters. The device-measured PA allowed for the examination of how PA levels in firefighters differentiate between on- and off-duty days. Additionally, the results demonstrated firefighters engaged in more activity while on-duty; however, this could be due to the increased wear-time of the accelerometer. The current study also monitored firefighters in their natural environment without restrictions to examine how PA intensity differences might occur on- versus off-duty.

The limitations to the current study were: (1) firefighters’ accelerometer data were only assessed during their awake hours for both on- and off-duty days. Firefighters wore the accelerometer for their entire 24 h shift, but only wore it during the hours they were awake when off-duty; therefore, the PA data could only be analyzed for waking hours; (2) only male firefighters participated, as there were only males employed at this particular fire department; (3) the participants were from the same department with the same tour format, and (4) the small sample size makes the data less generalizable to other departments. Although the sample size is relatively small, this is the first device-measured PA study that examined on- and off-duty firefighters in their natural environments. Accordingly, these results may not be transferrable to a department with different tour structures.

## 5. Conclusions

Based on the results of this study, and with the absence of published device-measured PA data in firefighters, we conclude that there is a need for increased PA levels in career, full-time firefighters while both on- and off-duty. The increased sedentary time, while on- and off-duty can potentially increase firefighters’ risk for developing underlying cardiovascular disease, and an increased risk of experiencing a sudden cardiac event when engaging in arduous emergency tasks. On average, firefighters in this study were more likely to meet ACSM guidelines on- compared to off-duty, which could be detrimental to their overall health and well-being as they spend three days of a nine-day tour on- compared to off-duty. Firefighters must rely on their health and physical fitness to adequately perform the physiologically demanding tasks during emergency calls. Future researchers need to continue to collaborate with fire departments across the country to assess ways to enhance PA levels in firefighters.

## Figures and Tables

**Table 1:** Dependent *t*-test values and effect sizes (*d*) for physical activity (PA) intensities.

PA Intensity	*t*	df	*p*	On-Duty M(SD)	Off-Duty M (SD)	*d*
Sedentary	0.46	28	0.650	563.31 (63.24)	555.25 (111.10)	0.09
% Sedentary	1.358	28	0.185	59.35 (6.06)	61.64 (10.20)	0.27
LPA	2.35	28	0.026	351.11 (59.90)	315.83 (86.90)	0.47
% LPA	−1.06	28	0.296	36.92 (5.50)	35.27 (9.67)	0.21
MPA	1.72	28	0.097	30.50 (17.48)	24.66 (14.94)	0.36
% MPA	−1.24	28	0.255	3.19 (1.80)	2.75 (1.61)	0.26
VPA	1.23	28	0.228	5.02 (7.41)	3.16 (6.65)	0.26
% VPA	−1.18	28	0.247	0.54 (0.80)	0.35 (0.74)	0.26
MVPA	2.00	28	0.055	35.51 (19.22)	27.82 (18.91)	0.40
% MVPA	−1.57	28	0.128	3.73 (2.00)	3.09 (2.03)	0.32

Mean (M) and SD are listed in minutes for PA intensities without a %.

**Table 2:** Step count categorization frequency.

Step Count Categorization	On-Duty Frequency (%)	Off-Duty Frequency (%)
<5000	1 (3.4)	4 (13.8)
5000–7499	3 (10.3)	10 (34.5)
7500–9999	12 (41.4)	11 (37.9)
>10,000	9 (31.0)	2 (6.9)
>12,500	4 (13.8)	2 (6.9)

**Table 3:** Independent samples *t*-test values and effect sizes (*d*) for on-duty PA intensities for firefighters meeting and not meeting American College of Sports Medicine (ACSM) guidelines.

PA Intensity	*t*	df	*p*	Met ACSM *n* = 17 M (SD)	Not Meet ACSM *n* = 12 M (SD)	*d*
Sedentary	1.64	27	0.112	547.58 (62.68)	585.60 (59.50)	0.62
% Sedentary	2.97	27	0.005	56.85 (5.57)	62.89 (5.00)	1.13
LPA	2.01	27	0.054	368.96(61.86)	325.81 (48.73)	0.77
% LPA	−1.59	27	0.123	38.25 (5.52)	35.04 (5.09)	0.60
MPA	4.15	27	0.000	39.50(17.15)	17.74(6.84)	1.67
% MPA	−4.07	27	0.000	4.10 (1.78)	1.89 (0.70)	1.53
VPA	2.39	27	0.025	7.37 (8.32)	1.70 (4.33)	0.85
% VPA	−2.17	27	0.039	0.79 (0.90)	0.18 (0.45)	0.81
MVPA	5.32	27	0.000	46.87(16.70)	19.44 (7.35)	2.13
% MVPA	−5.16	27	0.000	4.89 (1.78)	2.07 (0.75)	1.94

Mean (M) and SD are listed in minutes for PA intensities without a %.

**Table 4:** Independent samples *t*-test values and effect sizes (*d*) for off-duty PA intensities for firefighters meeting and not meeting ACSM guidelines.

PA Intensity	*t*	df	*p*	Met ACSM *n* = 9 M (SD)	Not Meet ACSM *n* = 20 M (SD)	*d*
Sedentary	−0.25	27	0.980	554.46 (101.95)	555.61 (117.53)	−0.01
% Sedentary	0.63	27	0.534	59.84 (9.39)	62.45 (10.68)	0.25
LPA	0.98	27	0.923	318.22 (83.09)	314.76 (90.65)	0.04
% LPA	0.27	27	0.792	34.55 (9.19)	35.60 (10.09)	0.11
MPA	7.57	27	0.000	42.70 (9.98)	16.54 (7.97)	2.90
% MPA	−6.74	27	0.000	4.61 (1.03)	1.91 (0.98)	2.71
VPA	2.75	27	0.025	9.22 (9.58)	0.43 (1.10)	0.85
% VPA	−4.02	27	0.000	1.01 (1.07)	0.05 (0.12)	1.62
MVPA	9.19	27	0.000	51.92 (11.93)	16.97 (8.23)	1.29
% MVPA	−8.41	27	0.000	5.62 (1.25)	1.95 (1.01)	3.38

Mean (M) and SD are listed in minutes for PA intensities without a %.
